# Combating Antibiotic Tolerance Through Activating Bacterial Metabolism

**DOI:** 10.3389/fmicb.2020.577564

**Published:** 2020-10-22

**Authors:** Yuan Liu, Kangni Yang, Haijie Zhang, Yuqian Jia, Zhiqiang Wang

**Affiliations:** ^1^College of Veterinary Medicine, Yangzhou University, Yangzhou, China; ^2^Institute of Comparative Medicine, Yangzhou University, Yangzhou, China; ^3^Jiangsu Co-innovation Center for Prevention and Control of Important Animal Infectious Diseases and Zoonoses, Yangzhou University, Yangzhou, China; ^4^Joint International Research Laboratory of Agriculture and Agri-Product Safety, The Ministry of Education of China, Yangzhou University, Yangzhou, China

**Keywords:** antibiotic resistance, antibiotic tolerance, antibiotic efficacy, bacteria, metabolism, metabolites

## Abstract

The emergence of antibiotic tolerance enables genetically susceptible bacteria to withstand the killing by clinically relevant antibiotics. As is reported, an increasing body of evidence sheds light on the critical and underappreciated role of antibiotic tolerance in the disease burden of bacterial infections. Considering this tense situation, new therapeutic strategies are urgently required for combating antibiotic tolerance. Herein, we provide an insightful illustration to distinguish between antibiotic resistance and tolerance, and highlight its clinical significance and complexities of drug-tolerant bacteria. Then, we discuss the close relationship between antibiotic tolerance and bacterial metabolism. As such, a bacterial metabolism-based approach was proposed to counter antibiotic tolerance. These exogenous metabolites including amino acids, tricarboxylic acid cycle (TCA cycle) metabolites, and nucleotides effectively activate bacterial metabolism and convert the tolerant cells to sensitive cells, and eventually restore antibiotic efficacy. A better understanding of molecular mechanisms of antibiotic tolerance particularly *in vivo* would substantially drive the development of novel strategies targeting bacterial metabolism.

## Introduction

Discovery and wide application of antibiotics save millions of lives in the past decades (Marston et al., 2016). Indeed, antibiotics are not only special drugs for infectious diseases, but also as an important cornerstone for the vigorous development of surgical medicine (Ramakrishna et al., 2014). The fortuitous discovery of penicillin by Fleming in 1929 offers an unprecedented regimen for infections caused by Gram-positive bacteria such as *Staphylococcus aureus*, and the identification of streptomycin in 1943 enables the effective control of tuberculosis by *Mycobacterium tuberculosis*. As such, these previous endeavors paved the way for the coming of the golden era of antibiotics. However, just as the saying goes, “*Where there is oppression, there is resistance*”, bacteria have evolved versatile means to antagonize antibiotic killing, such as antibiotic resistance (Aminov, 2009). Antibiotic resistance is commonly conferred by the emergence of resistance genes in bacteria, and could spread by plasmid-mediated horizontal transfer during inter‐ and intra-species. However, it was increasingly observed that bacteria could survive under extensive antibiotic treatments without genotypic changes. This phenomenon was termed antibiotic tolerance, which was first described by Tomasz et al. (1970). To be specific, they depicted *Streptococcus pneumoniae* with a deficient autolytic system protecting the pathogen from lysis by penicillin. Recently, growing evidence demonstrated that antibiotic tolerance profoundly diminishes antibiotic efficacy both *in vitro* and in clinic (Handwerger and Tomasz, 1985; Fridman et al., 2014). Tolerant bacteria are phenotypically resistant but genetically susceptible to antibiotic treatment (Wiuff et al., 2005; Windels et al., 2019). Traditional testing measures for antibiotic resistance such as minimum inhibitory concentration (MIC) assay (Andrews, 2001) and PCR analysis are not applicable for the detection of antibiotic tolerance. As a consequence, antibiotic tolerance is generally overlooked in clinical practice, existing as a “hidden bomb” that threatens antibiotic efficacy and human health worldwide. Besides, it has been suggested that antibiotic tolerance facilitates the emergence and evolution of antibiotic resistance (Levin-Reisman et al., 2017).

Thus far, several strategies have been proposed for addressing the antibiotic resistance crisis, including novel antibiotics, combinations therapy, and other alternatives to antibiotics. However, these therapies are limited for drug-resistant bacteria but not effective against tolerant bacteria. As such, there is an urgent need to mine distinct strategies to combat antibiotic tolerance, not only for improving treatment outcomes but also to address the crisis caused by antibiotic resistance at the root. Given that phenotypic tolerance is highly sensitive to environmental conditions, which either directly interfere with antibiotics or alter bacterial physiology, thus we reasoned that bacterial metabolic networks-based strategies might be a feasible approach to counteract antibiotic tolerance.

In this review, we first systematically compare the difference between antibiotic resistance and tolerance. Subsequently, we discuss the close relationship between bacterial metabolism and antibiotic tolerance. Based on this, bacterial metabolic network-based approaches were proposed to deal with antibiotic tolerance, by which bacterial metabolic state was re-activated. These findings suggest that bacterial metabolism regulators can serve as potential antibiotic adjuvants, thus providing a distinctly different perspective in the fight against antibiotic tolerance.

## Distinguishing Between Antibiotic Resistance and Tolerance

**Figure 1:**
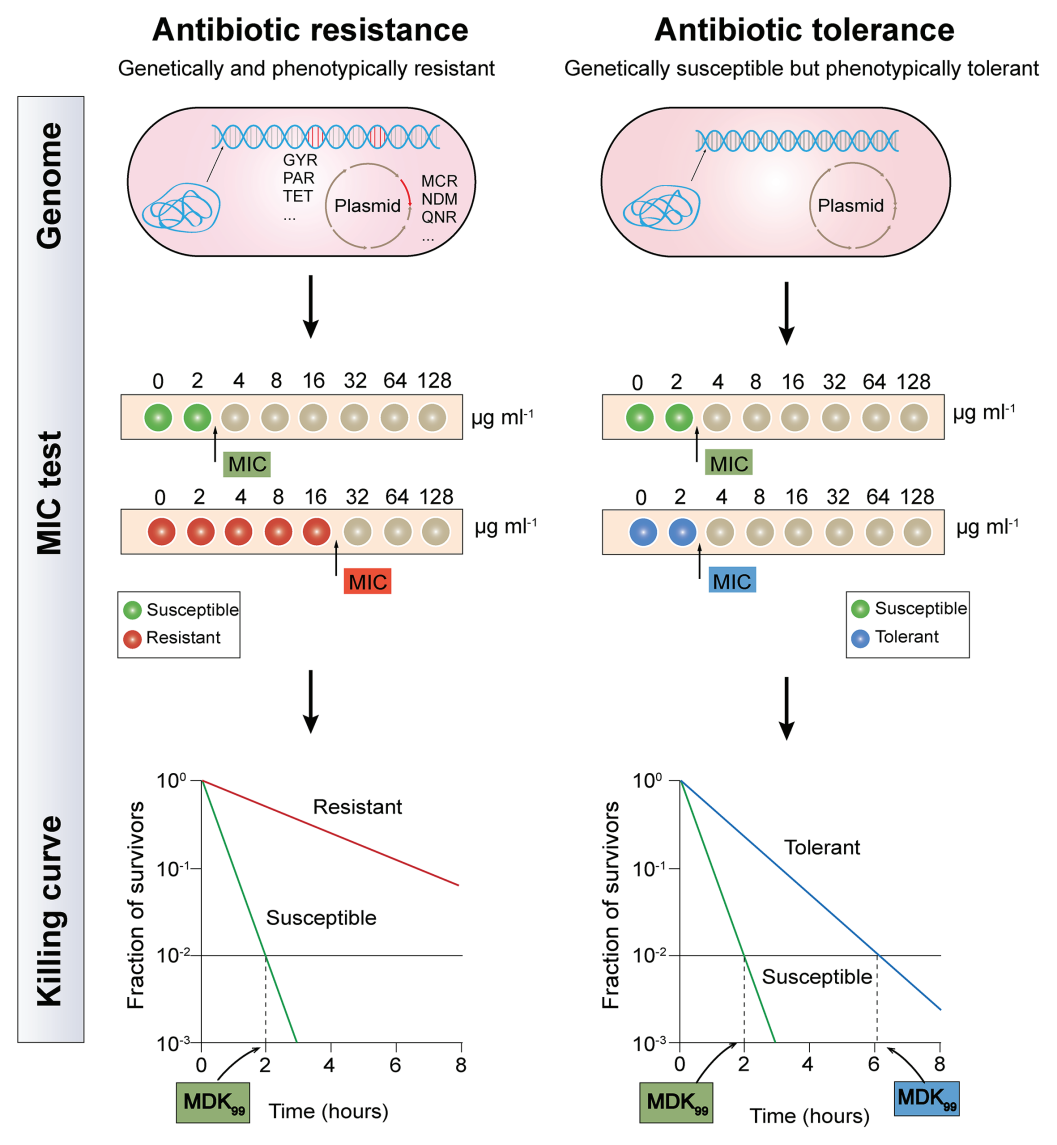
Distinguishing between antibiotic resistance and antibiotic tolerance (Brauner et al., 2016). Antibiotic resistance (the left half of diagram) is characterized by both bacterial genetic and phenotypic resistance; the MIC for a resistant bacteria strain is substantially higher than the MIC for a susceptible bacteria strain; the minimum duration for killing for 99% bacterial cells (MDK_99_) for a resistant bacteria strain is substantially higher than the MDK_99_ for a susceptible bacteria strain. Antibiotic tolerance (the right half of diagram) is characterized by genetic sensitivity but phenotypic tolerance; the MIC for a tolerant bacteria strain is similar to the MIC for a susceptible bacteria strain; MDK_99_ for a tolerant bacteria strain is 3-fold higher than the MDK_99_ for a susceptible bacteria strain.

Although both antibiotic resistance and tolerance provide a surviving advantage for bacteria after exposure to antibiotics, they are utterly different biology phenomena (Figure 1). Resistance is the inheritable capability of microbes to survive high levels of antibiotics and can be determined by the MIC test (Mouton et al., 2018). Be more specific, higher concentrations of antibiotics are required for resistant bacteria when to act as effectively as sensitive bacteria. Tolerance, on the one hand, is more widely used to describe the ability of microorganisms to be briefly exposed to high levels of antibiotics but displaying unaltered MIC values, whether inherited or not (Brauner et al., 2016). On the other hand, their underlying molecular mechanisms are different. The majority molecular mechanisms of antibiotic resistance have been identified through the ongoing study of antibiotic-resistant bacterial strains, including mutations of drug target, deactivation of antibiotic by hydrolases or modified enzyme, and decreased intracellular antibiotic accumulation due to reduced permeability or over-activation of efflux pumps (Blair et al., 2015). These resistance determinants are commonly conferred by resistance genes that are located on chromosomes or mobilized plasmids. For example, metallo-β-lactamases (MBLs) such as NDM-1 could hydrolyze carbapenems in a Zn^2+^-dependent manner (Walsh et al., 2011), whereas Tet(X) and its variants selectively hydroxylate the tigecycline at C11a and confer high-levels of tigecycline resistance (He et al., 2019; Sun et al., 2019). Besides, mobile colistin resistance (*mcr*)-encoded phosphoethanolamine transferase catalyzes the addition of the cationic moiety to the phosphate groups of lipopolysaccharides (LPSs; Liu et al., 2016), resulting in decreased affinity between colistin and LPS. By contrast, antibiotic tolerance refers to genetically susceptible bacteria but phenotypically tolerant to antibiotic killing (Liu et al., 2019b). Typically, bacterial metabolism-mediated tolerance can be identified in two types: tolerance by “slow growth” or “by lag” (Fridman et al., 2014; Brauner et al., 2016). Slow growth-mediated tolerance occurs at a steady-state (Thonus et al., 1982), yet tolerance by lag is a transient state owing to stressful or starvation conditions (Fridman et al., 2014). Notably, antibiotic tolerance can either be inherited such as an inherently slow growth rate in specific strain, or noninherited under the circumstance of poor growth conditions or within biofilms or host cells (Kitano and Tomasz, 1979; Bernier et al., 2013).

It has been proved that tolerance applies only to bactericidal antibiotics, rather than bacteriostatic antibiotics. Because all bacteria are expected to survive after short-term exposure to bacteriostatic antibiotics, which will not be fatal, but only prevent bacterial growth (Ocampo et al., 2014). As tolerant bacteria and non-tolerant bacteria have the same MIC value, the MIC test is not applicable to evaluate antibiotic tolerance. Although minimum bactericidal concentration (MBC, the antibiotic concentration that is required to kill ≥99.9% of bacterial cells) and MBC/MIC ratio were also proposed as a measure of antibiotic tolerance, these metrics are only reliable for drug-induced tolerance but correlate poorly with other types of tolerance (Keren et al., 2004; Pasticci et al., 2011). Recently, a quantitative indicator based on the time-kill curves, which is called the minimum duration for killing (MDK; Brauner et al., 2017), has been designed to eliminate a certain percentile of the bacterial population. Tolerant bacteria and susceptible strain usually have a similar MIC value, but the MDK_99_ (the minimum duration for killing for 99% bacterial cells) for a tolerant strain is remarkably higher than that in a susceptible strain (Balaban et al., 2019). Nevertheless, antibiotic tolerance has not been fully characterized, owing to the lack of definite quantitative indicators.

Phenotype tolerance to antibiotic treatment exacerbates the lack of effective antibacterial therapy against chronic infections (Fauvart et al., 2011). Based on the mechanism of antibiotic resistance, traditional methods of dealing with drug-resistant bacteria act on critical biosynthesis processes such as membrane synthesis, DNA replication and transcription, and protein synthesis (Liu et al., 2019a), which have limited efficacy in the fight against tolerant bacteria. Thus, novel strategies are urgently needed to combat tolerant bacteria.

## Correlation Between Antibiotic Tolerance and Bacterial Metabolism

As prior mentioned, “tolerance by slow growth” and “tolerance by lag” have been identified as two representative mechanisms for tolerance formation, which are both ultimately attributed to changes in the metabolic status of bacteria (Brauner et al., 2016). In other words, reduced metabolism is one of the crucial drivers for the emergence of antibiotic tolerance. Metabolically dormant bacteria constitute a growing threat to many effective treatment regimens, the treatment of *Pseudomonas aeruginosa* with aminoglycosides was taken as an example, the bactericidal activity of aminoglycosides requires proton motive force (PMF)-dependent transport to allow sufficient cell penetration (Allison et al., 2011). When the central carbon metabolism is perturbed by the addition of carbon source metabolites, the sensitivity of *P. aeruginosa* to tobramycin also changed. In these metabolites, glyoxylate acts as an inducer of antibiotic tolerance by retarding TCA cycle activity and cellular respiration, thereby decreasing transmembrane PMF and drug internalization (Meylan et al., 2017). Similarly, in the study of antibiotic tolerance in *M. tuberculosis* (Mtb), isocitrate lyase mediates broad antibiotic tolerance by remodeling Mtb’s TCA cycle, including increased glyoxylate shunt activity and suppressed TCA cycle (Nandakumar et al., 2014). Consistent with this observation, another quantitative proteomics analysis also demonstrated that the decrease in energy metabolism and central carbon are associated with levofloxacin tolerance in naturally occurring waterborne pathogen *Vibrio alginolyticus* (Cheng et al., 2018).

**Figure 2:**
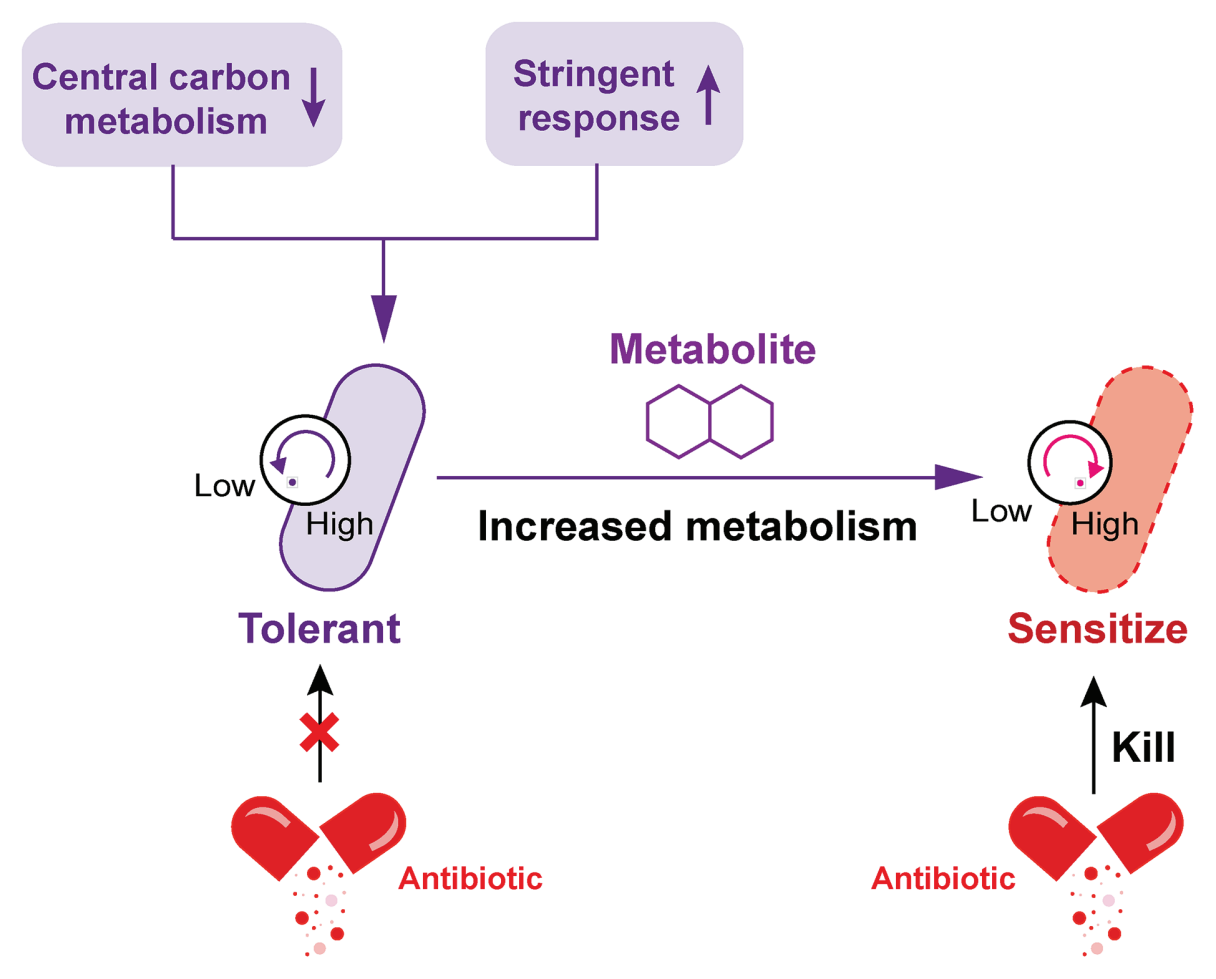
Increased metabolism restores the susceptibility of tolerant bacteria to antibiotics. The decrease of central carbon metabolism level and activation of stringent response drive the inhibition of bacterial metabolic state, which leads to the formation of antibiotic tolerance. By contrast, the addition of specific exogenous metabolites is able to improve the bacterial metabolic state; tolerant bacteria were subsequently converted to metabolically active bacteria and restore susceptibility to antibiotic killing.

Marked antibiotic tolerance can also be produced by a metabolic downshift coinciding with nutrient deprivation (Braeken et al., 2006; Traxler et al., 2008). When bacteria are exposed to nutrient constraints, they respond by activating a strict response (SR) that changes their state (Boutte and Crosson, 2013), in this regard, the bacterial protein synthesis and other metabolic activities are remarkably shut down. By way of example, *P. aeruginosa* in a nutritionally restricted environment showed tolerance to multiple antibiotics owing to the activation of SR (Nguyen, 2011; Khakimova et al., 2013; Martins et al., 2018). Besides, a recent study investigated the relative contribution of growth rate and metabolic state of bacteria to antibiotic lethality (Lopatkin et al., 2019). As a result, they found that bacterial metabolic state was more correlated with the antibiotic lethality than growth rate. Similarly, another study demonstrated that the lethal mechanism of beta-lactams is not simply inhibiting penicillin-binding proteins (PBPs), they result in futile cycling of cell wall synthesis and degradation, suggesting that dysfunction of bacterial metabolism is related to antibiotic lethality (Cho et al., 2014b). Collectively, these examples suggest that antibiotic tolerance is closely associated with low levels of metabolism in bacteria, thus it may be possible to restore the sensitivity of tolerant bacteria to antibiotics by altering the metabolic state of bacteria (Figure 2).

## Bacterial Metabolism-Based Strategies Against Antibiotic Tolerance

Since the metabolic state of bacteria correlates with their susceptibility to antibiotic treatment, instead the development of new drugs or adjuvant strategies, modulating bacterial metabolic activity provides a universal strategy to potentiate bactericidal antibiotic killing (Stokes et al., 2019). The metabolic state of a bacterium is closely related to its environment including nutrients, so it is a proposed method to change the metabolic state and restore antibiotic sensitivity by adding exogenous metabolites such as amino acids, tricarboxylic acid (TCA) cycle metabolites and nucleotides during bacterial growth.

### Amino Acid Supplementation

**Figure 3:**
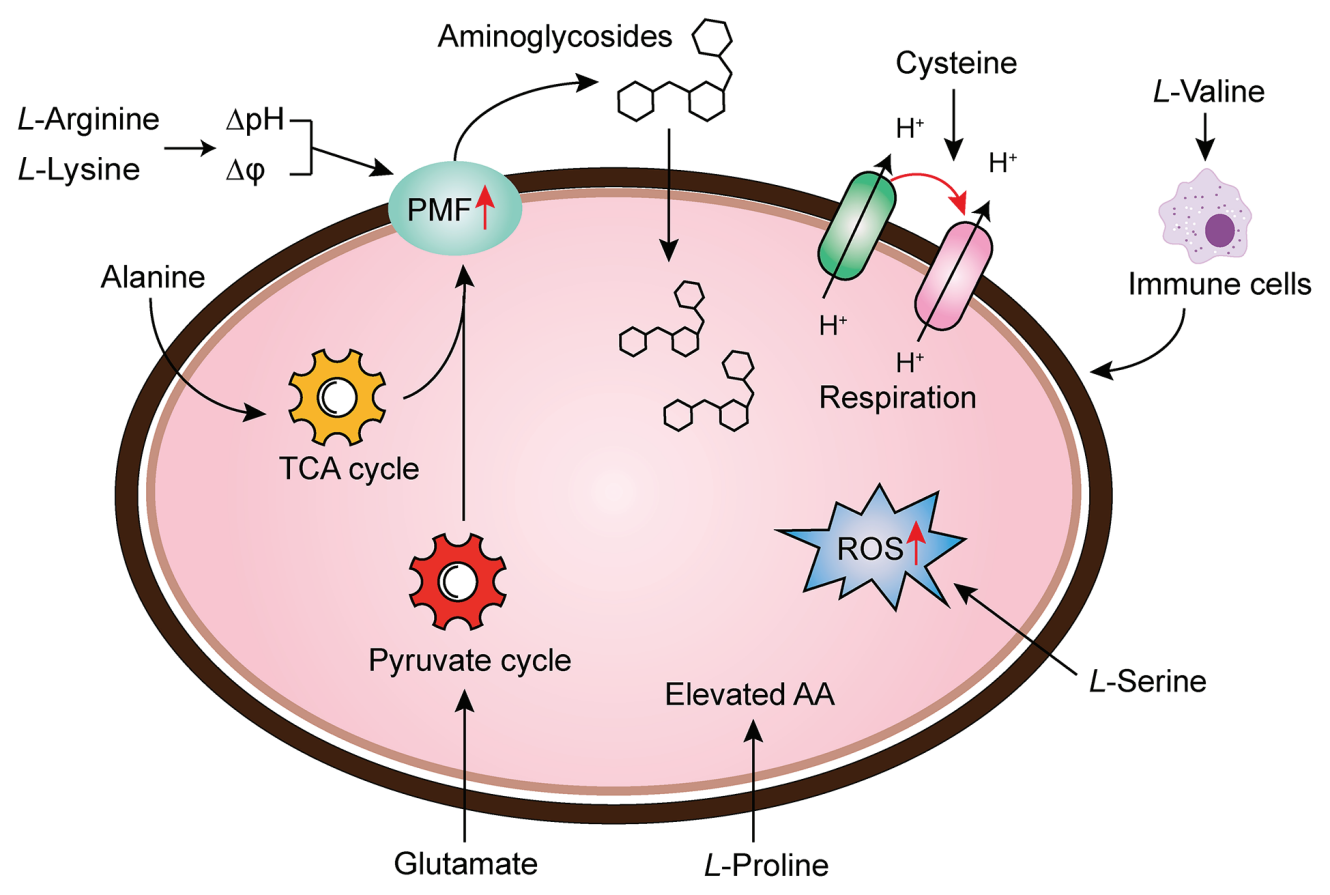
Amino acid supplementation reverses antibiotic tolerance (Liu et al., 2019b). Potentiation of antibiotic efficacy against antibiotic tolerance by exogenous amino acid supplementation results from enhanced amino acid metabolism, proton motive force (PMF), reactive oxygen species (ROS), bacterial cellular respiration, or host immunity.

Amino acids are one of the most active macromolecules in the construction of living organisms and are the basic materials for the construction of cells and the repair of tissues (Wu, 2013). It has been reported that the combination of multiple amino acids and antibiotics can reinforce antibiotic activity through increasing PMF, upregulating pyruvate cycle (P cycle) or stimulating bacterial respiration, the production of reactive oxygen species (ROS), or host immune response (Figure 3).

There are a series of evidence indicate that increasing the transmembrane PMF stimulates the internalization of aminoglycoside antibiotics (Allison et al., 2011; Radlinski et al., 2019). In particular, the PMF is the sum of two components: the electric potential (△ψ) and the transmembrane proton gradient (△pH; Farha et al., 2013). It has been widely acknowledged that the uptake of aminoglycoside is mainly dependent on △ψ, whereas uptake of tetracyclines is driven by △pH (Taber et al., 1987; Stokes et al., 2020). For example, fructose or mannitol was found to stimulate the production of PMF *via* increasing △ψ (Allison et al., 2011). Lebeaux et al. found that the bactericidal activity of aminoglycosides against antibiotic-tolerant *Escherichia coli* could be reversed through the supplementation with the basic amino acid, *L*-arginine, which results in increased environmental pH and PMF, as well as the uptake of drugs (Lebeaux et al., 2014). Controversially, differing from the above notion, they demonstrated that the effect of *L*-arginine on gentamicin against bacterial infection probably relies on transmembrane △pH rather than △ψ. Furthermore, the beneficial effect of *L*-arginine on aminoglycosides against various pathogens has been verified in both *in vitro* and *in vivo* rat models. Similarly, another basic amino acid *L*-lysine was also found to sensitive both Gram-negative bacteria (*Acinetobacter baumannii*, *E. coli*, and *Klebsiella pneumoniae*), and a Gram-positive bacterium (*Mycobacterium smegmatis*) to aminoglycosides *via* promoting the transmembrane proton gradient (ΔpH), which in turn enhances PMF and stimulates the uptake of aminoglycosides (Deng et al., 2020). Despite the mechanistic difference in basic amino acids and other metabolites, an increase in PMF is of great importance for the restoration of aminoglycosides activity.

In bacteria, the PMF results from the extrusion of protons by the electron transport chain are highly dependent on the electron donor such as NADH, which produced from the TCA cycle. Therefore, activation of the TCA cycle is also crucial for the bactericidal activity of aminoglycoside antibiotics. In 2015, by comparing the metabolome differences between kanamycin-resistant *Edwardsiella tarda* LTB4 and wild-type strains, Peng et al. (2015) revealed that the alanine and glucose abundance significantly decreased in resistant bacteria. Inspired by this unique phenomenon, they investigated whether kanamycin-resistant *E. tarda* LTB4 cells would restore their sensitivity to aminoglycoside antibiotics when cultured with exogenous alanine and/or glucose. Subsequent studies indicated that the addition of exogenous alanine and/or glucose activated the TCA cycle of the tolerant bacteria and enhanced PMF, thereby increasing their sensitivity to kanamycin, both *in vitro* and in a mouse model of urinary tract infection (Peng et al., 2015). Following this line of thought, the author further showed that glutamate, another depressed biomarker, also promoted the inactivation of drug-resistant bacteria by kanamycin. Mechanical studies indicated that exogenous glutamate reverted the phenotype of antibiotic resistance in both *E. tarda* and *E. coli* by modulating flux through the P cycle, emphasizing that the P cycle is a common pathway of respiration and energy production. More specifically, these results robustly implied that strengthening the P cycle is conducive to respiration and metabolism of bacteria (Su et al., 2018).

To the best of our knowledge, the treatment of Tuberculosis (TB) caused by the bacillus Mtb is a lengthy and arduous process (approximately six mouth), which majorly due to the presence of a small population of tolerant bacteria (Dheda et al., 2016; Furin et al., 2019). Meanwhile, this process may result in the emergence of multidrug-resistant (MDR) TB, which aggravates the infections inevitably (Lobue and Mermin, 2017). Thus, a shorter treatment option would be beneficial (Tiberi et al., 2018). A recent study revealed that the upregulation of bacterial cellular respiration enhanced the antibiotic susceptibility to Mtb cells (Vilcheze et al., 2017). They showed that the addition of cysteine can enhance the effect of isoniazid killing against exponentially growing Mtb cells, and prevent the emergence of drug-resistant mutants. In-depth investigations showed that small thiols could shift the menaquinone/menaquinone balance toward a reduced state, thus stimulating Mtb respiration and potentiating killing by isoniazid or rifampicin. Also, the INH/thiol combination was more effective both *in vitro* and infected murine macrophages by Mtb compared with monotherapy. Additionally, the authors also found that ROS concentrations were higher in INH/Cys-treated Mtb leading to DNA damage, although cysteine is not primarily ROS-mediated as a potentiator, the involvement of ROS production indeed correlates with some specific antibiotic killing. In agreement with this observation, when *L*-serine interacts with fluoroquinolones such as ofloxacin or moxifloxacin in Gram-negative bacteria, the bacteria can produce increased NADH and interfere with Fe-S clusters related to the Fenton’s reaction, thereby stimulating the over-production of endogenous ROS and ultimately enhancing the bactericidal action of drugs (Duan et al., 2016). Apart from these mechanisms, the immune modulation of amino acids to host cells has also been revealed. To illustrate, the metabolomic analysis of *K. pneumonia* infected mice showed that *L*-valine was a key metabolite to promote the survival of mice challenged by *K. pneumoniae*; moreover, this similar effect could be observed with other Gram-negative pathogens such as *E. coli*, *P. aeruginosa*, or MRSA, further implying that *L*-valine may be one of the metabolites that regulate immune functions (Chen et al., 2017). It has also been verified that the addition of exogenous *L*-proline has a significant impact on the mortality of tilapia infected by *Streptococcus agalactiae* at higher water temperatures, among which the addition of exogenous *L*-proline can enhance the anti-infection ability of tilapia through triggering its immune response (Zhao et al., 2015). Collectively, these findings provide new insight into the unique functions of amino acids as metabolic modulators in reverting antibiotic tolerance.

### TCA Related Metabolites Supplementation

**Figure 4:**
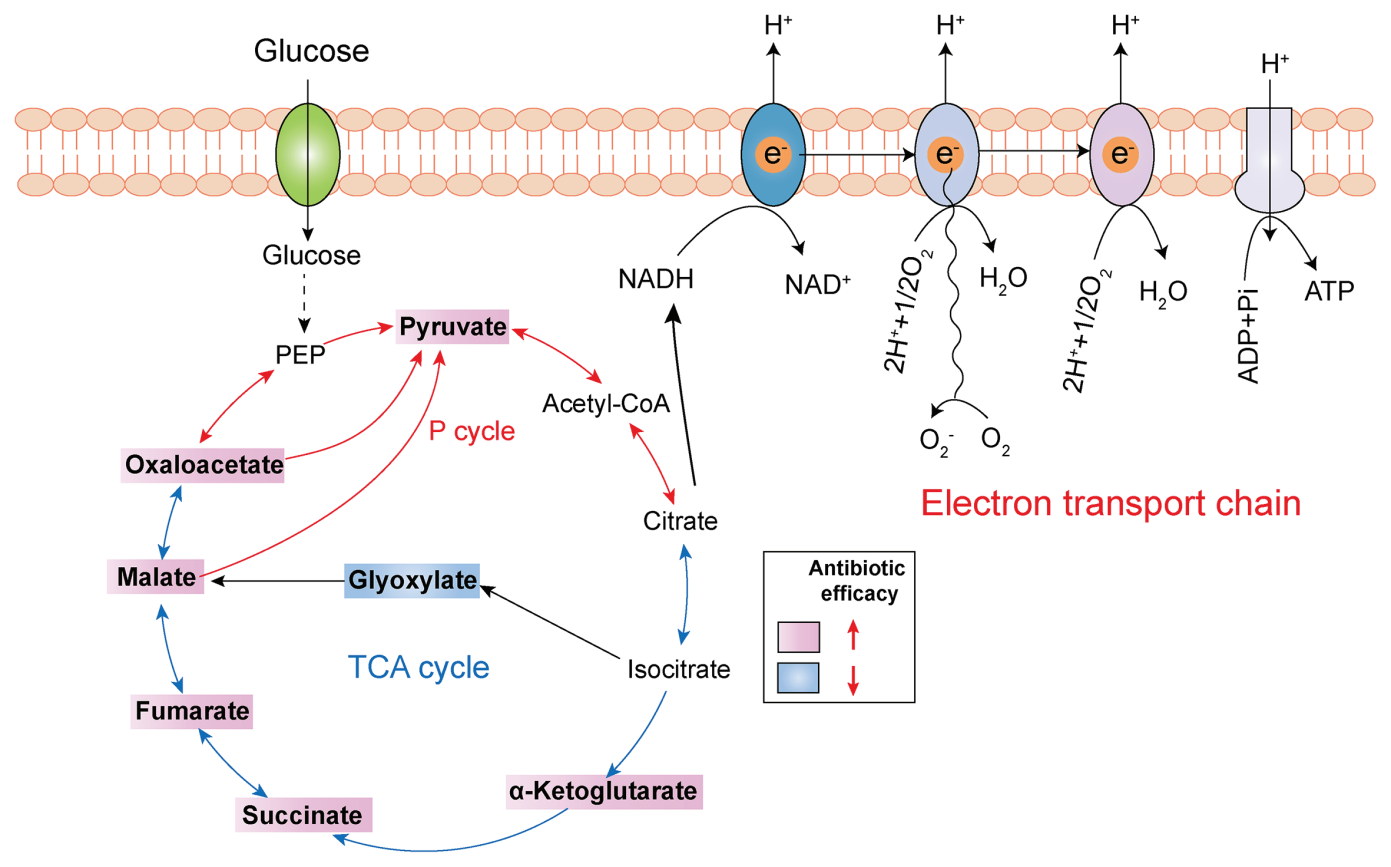
Tricarboxylic acid (TCA) cycle metabolites supplementation restores antibiotic efficacy. The addition of exogenous TCA metabolites (pink background) facilitates pyruvate cycle (red line) or TCA cycle (blue line) and upregulates bacterial electron transport chain, thus improving antibiotic efficacy. However, glyoxylate supplementation (blue background) inhibits TCA cycle and downstream respiration, thereby triggering antibiotic tolerance.

The TCA cycle, as the common and ultimate metabolic pathway of the three major nutrients, and the hub of the metabolism of carbohydrate lipid and amino acid, is an integral part of the metabolism of bacteria (Salway, 2018). Recently, a series of studies revealed that boosting the TCA cycle could alter the metabolic state of bacteria, and thereby improving antibiotic efficacy (Figure 4). In 2017, Meylan et al. found that when *P. aeruginosa* was co-cultured with carbon source metabolites in different central metabolic pathways, there were significant differences in the bactericidal activity of tobramycin, implying that the sensitivity of *P. aeruginosa* to tobramycin was related to the disorder of central carbon metabolism. Consistently, lower TCA cycle metabolites such as fumarate, succinate, α-ketoglutarate, and pyruvate significantly sensitized stationary-phase cells to tobramycin. Mechanistic investigations showed that fumarate activated cellular respiration and generated more PMF by stimulating the TCA cycle as a tobramycin potentiator, which was consistent with the prior findings that aminoglycosides’ sensitivity is associated with PMF and drug internalization levels (Meylan et al., 2017). By contrast, glyoxylate-treated cells bypass the generation of two reducing equivalents (NADH and FADH_2_), thereby displaying a lower TCA cycle and decreased cellular respiration, which eventually lead to decreased antibiotic efficacy. In 2018, Su et al. found that glutamate promoted the inactivation of drug-resistant bacteria by kanamycin, and the underlying mechanism revealed a previously unknown prevalent pathway termed P cycle (Su et al., 2018). In the further study of the P cycle, excess carbon sources such as oxaloacetate and pyruvate also improved kanamycin’s lethality to *E. tarda* through the same mechanism, indicating that P cycle is a vital pathway for bacterial respiration and energy production.

Another example is quinolone antibiotics, which kill pathogens by targeting the DNA of bacteria, hindering the DNA cyclotron enzyme, further causing the irreversible damage of bacterial DNA (Mitscher, 2005; Kottur and Nair, 2016; Mohammed et al., 2019). There are also studies showing that the bactericidal effect of quinolones is affected by the density of the cell population, which is called density-dependent persistence (DDP). As the consumption of certain metabolites, such as carbon catabolism and oxidative phosphorylation, has been implicated in DDP, the supplementation of glucose and appropriate terminal electron receptors (such as fumarate) in stationary-phase cultures sensitized cells to quinolone killing by stimulating respiratory metabolism (Gutierrez et al., 2017).

In addition to *in vitro* experimental studies, *in vivo* assay also showed that the benefit of elevated TCA cycle in preventing animal infections. For instance, exogenous malate was confirmed to boost the TCA cycle and increase the enzymatic activities of α-ketoglutaric dehydrogenase (KGDH) and succinate dehydrogenase (SDH; Yang et al., 2018, 2020). Consequently, increased survival of zebrafish to *Vibrio alginolyticus* infection was observed. Besides, a recent study utilized reprogramming metabolomics to explore the metabolic mechanisms of colistin resistance in *V. alginolyticus*. They found that colistin-resistant *V. alginolyticus* was characterized by decreased central carbon metabolism and energy metabolism. By contrast, the addition of metabolites such as pyruvate reverted colistin activity against resistant *V. alginolyticus* both *in vitro* and in zebrafish (Li et al., 2020).

### Nucleotide Supplementation

Nucleotides, as one of the essential metabolites of organisms, are widely distributed in the human body and have a variety of biological functions, such as constituting nucleic acid, storeing energy, delivering drugs, and participating in metabolism and physiological regulation (Poijärvi-Virta, 2006; Cho et al., 2014a; Roy et al., 2016). As mentioned earlier, the lethality of bactericidal antibiotics in bacteria correlates with altered bacterial metabolism, including the increased abundance of carbon metabolites in the center of cells and the disruption of nucleotide pools. Accumulated observations imply that the dysfunction of nucleotide metabolism is also associated with bactericidal antibiotic activity, regardless of their macromolecular targets. For example, a study manifested that the killing of β-lactams and quinolones is predominantly elicited by specific oxidation of the guanine to 8-oxyguanine in the nucleotide pool and its subsequent activity in nucleic acid transactions, leading to the overproduction of DinB (DNA polymerase IV) in *E. coli*, which eventually leads to bacterial death (Foti et al., 2012). Besides, the oxidation of deoxynucleotide cytosine triphosphate (dCTP) was also found to lead to antibiotic lethality against stationary-phase *mycobacteria* (Fan et al., 2018).

**Figure 5:**
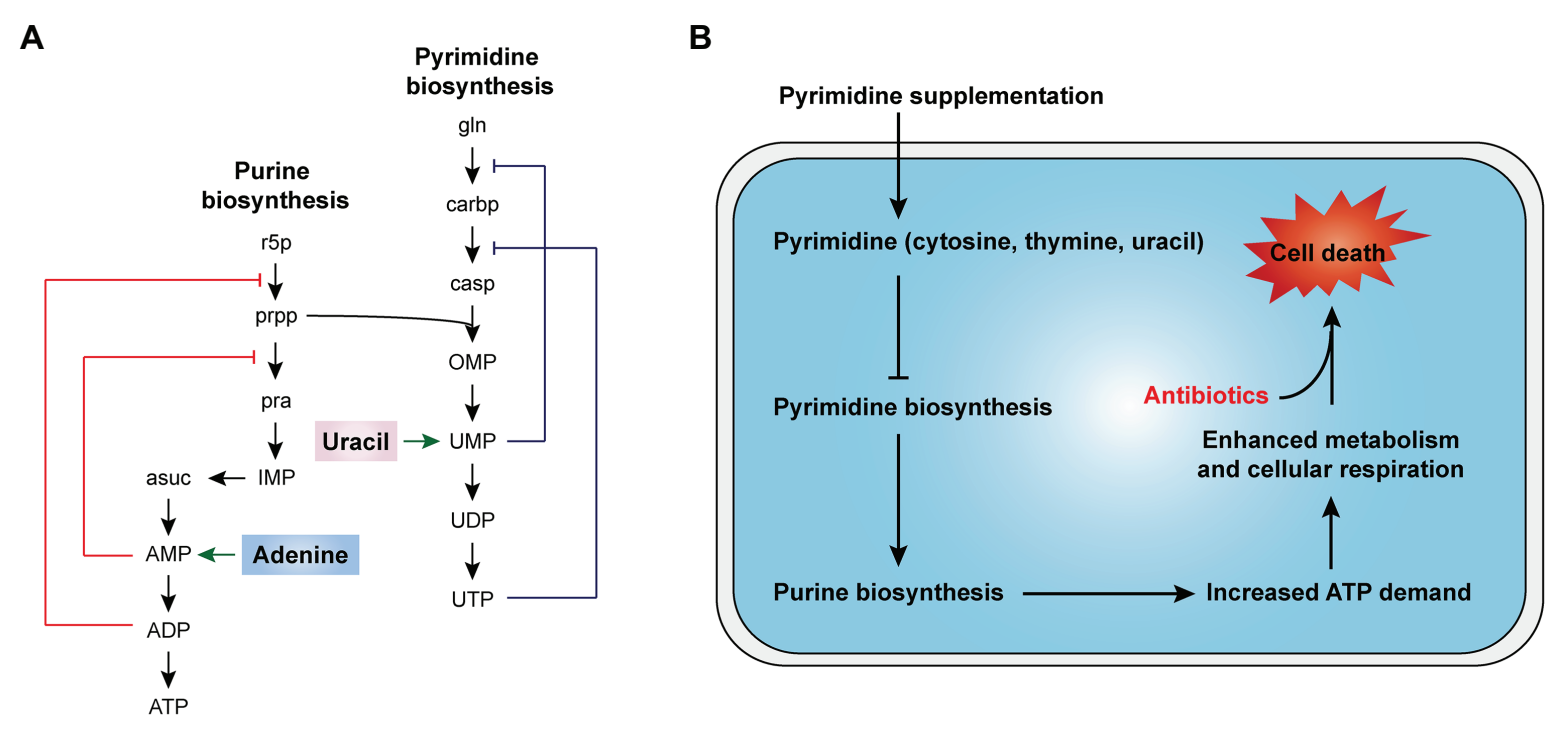
Nucleotides supplementation activates bacterial metabolism (Yang et al., 2019). **(A)** Purine and pyrimidine biosynthesis pathways and feedback inhibition by products. **(B)** The addition of exogenous pyrimidine (cytosine, thymine, and uracil) displays a feedback inhibition on the pyrimidine biosynthesis and triggers purine biosynthesis, thus increasing ATP demand, which drives increased activity through central carbon metabolism and cellular respiration, ultimately restoring the killing of antibiotics against tolerant bacteria.

In cells, nucleotides synthesis is made up of purine and pyrimidine synthesis pathway. On the basis of 5-phosphoribosyl pyrophosphate (PRPP), the *de novo* pathway enzymes produce purine and pyrimidine nucleotides through scratch simple molecules such as CO_2_ and amino acids (West, 2014; Lane and Fan, 2015). Meanwhile, the nucleotide end products would produce internal feedback inhibition on nucleotide biosynthesis pathways (Figure 5A). In 2019, Yang et al. develop a “white-box” machine-learning approach combining biochemical screening and network modeling to explore metabolic mechanisms of antibiotic lethality and to provide novel mechanistic insights. These results showed that adenine supplementation inhibited the biological activity of purines, reduced the demand for ATP and the metabolic activity of central carbon, and thus reduced the lethality of antibiotics. In contrast, pyrimidine such as uracil supplementation inhibited pyrimidine biosynthesis and in turn promoted purine biosynthesis pathway by PRPP accumulation and, consequently, increased antibiotic lethality (Figure 5B; Yang et al., 2019). Consistently, our recent study demonstrated that thymine can potentiate killing by bactericidal antibiotics against multiple Gram-negative bacteria *via* upregulating bacterial metabolism (Unpublished). Given those results, it is probable that pyrimidines may potentially serve as novel antibiotic adjuvants by promoting purine biosynthesis and altering the metabolic status of bacteria.

## Conclusion and Prospects

Antibiotic tolerance plays an important but underappreciated role in the evolution of antibiotic resistance. However, at present, there are no standard methods to distinguish between antibiotic tolerance and resistance accurately. Therefore, there is a strong need to deepen mechanisms of action of antibiotic tolerance and identify feasible coping strategies. A collection of studies on the tolerance formation mechanism demonstrates that it is tightly related to the metabolic state of bacteria. The primary manifestation is that antibiotics act on the important metabolic pathway of bacteria, thus changing the metabolic state of bacteria. In turn, the metabolic state of bacteria will also affect the efficacy of antibiotics. Inspired by this notion, we hypothesized that the addition of exogenous compounds to alter the metabolic state of bacteria might recover their sensitivity to antibiotics. As expected, the feasibility and effectiveness of this approach have been partially proved. It has been suggested that various exogenous amino acids, TCA cycle metabolites, and nucleotides can enhance the efficacy of bactericidal antibiotics through activating bacterial metabolism. Besides, other metabolites that enter upper glycolysis such as glucose, mannitol, and fructose could also potentiate antibiotic activity (Allison et al., 2011; Peng et al., 2015; Zeng et al., 2017). Meanwhile, some exogenous compounds have been found to modulate bacterial metabolism and thereby affect antibiotic activity. For example, artemisinin and structural analogs can improve the therapeutic effect of isoniazid on *M. tuberculosis* by inhibiting the dormancy pathway (Zheng et al., 2017). As such, the utilization of these exogenous metabolites as potential antibiotic adjuvants is an emerging and scalable strategy for developing novel antibiotic therapies. In this review, we distinguish the difference between antibiotic tolerance and drug resistance, and discuss the relationship between antibiotic tolerance and bacterial metabolism. Meanwhile, we provide an overview of strategies to restore bacterial sensitivity to antibiotics based on the metabolic network for existing tolerant bacteria according to different metabolite types.

However, the current researches on improving antibiotic efficacy based on the bacterial metabolic network also have some limitations. First, although the metabolic state of bacteria is a uniform characteristic defining antibiotic efficacy across in various physiologic states, the same metabolites did not increase the sensitivity of each pathogen, it is important to note that these strategies must be tuned on a pathogen-by-pathogen basis to be successful. For example, the sensitivity of aminoglycoside to *E. coli* can be enhanced with the addition of four metabolites (glucose, fructose, mannitol, and pyruvate), but fructose was the only one that increased the efficacy of gentamicin against tolerant *S. aureus* (Allison et al., 2011), suggesting that we should consider the characteristics of different pathogens in the process of screening and verifying metabolites that can be used as adjuvant of antibiotics to find species-specific therapeutic strategies. Another serious limitation is that most of the studies targeting tolerance have been proved *in vitro*, with only a few studies that have tested the strategy of using metabolites as antibiotic adjuvants *in vivo* animal models, whereas *in vivo* trials are essential for the application of new antibiotic strategies to actual clinical treatment. Notably, the dynamic changes of metabolites in the physiological environment of resistant bacterial infections cannot be obtained by *in vitro* experiments alone (Yang et al., 2017). It is important to note that several studies have shown that engineered bacterial sensor strains can be used to characterize *in vivo* infection dynamics and to verify the therapeutic effect of antibiotics (Certain et al., 2017). These sensor strains can be customized to detect and report on an array of environmental conditions, bacterial stress responses, and growth behaviors. This progress is worthy of further exploration for the *in vivo* efficacy of metabolites as novel antibiotic adjuvants.

Despite the gaps in our search for new approaches to reverse antibiotic tolerance by metabolite-based approaches, these findings discussed in this review provide a variety of strategies and ideas for exploring novel therapeutic regimens for combating antibiotic tolerance, and in future studies, the complex metabolic networks of bacteria should be more fully utilized.

## Author Contributions

All authors listed have made a substantial, direct, and intellectual contribution to the work, and approved it for publication.

### Conflict of Interest

The authors declare that the research was conducted in the absence of any commercial or financial relationships that could be construed as a potential conflict of interest.
